# Febuxostat and Cardiovascular Events: A Systematic Review and Meta-Analysis

**DOI:** 10.1155/2019/1076189

**Published:** 2019-02-03

**Authors:** John A. Cuenca, Javier Balda, Ana Palacio, Larry Young, Michael H. Pillinger, Leonardo Tamariz

**Affiliations:** ^1^Universidad Catolica de Santiago de Guayaquil, Ecuador; ^2^Division of Population Health and Computational Medicine, Miller School of Medicine, University of Miami, Miami, FL, USA; ^3^Veterans Affairs Medical Center, Miami, FL, USA; ^4^Division of Rheumatology, University of Miami, USA; ^5^Division of Rheumatology, New York University School of Medicine, USA; ^6^Veterans Affairs Medical Center, New York, NY, USA

## Abstract

**Background:**

Febuxostat is approved in the United States for the management of hyperuricemia in patients with gout. In November 2017 the FDA released a warning alert on a possible link between febuxostat and cardiovascular disease (CVD) reported in a single clinical trial.

**Objective:**

To conduct a systematic review and meta-analysis and assess the risk of major adverse cardiovascular events (MACE) in patients receiving febuxostat compared to a control group.

**Methods:**

We searched the MEDLINE and EMBASE database for studies published up until March 2018. We included randomized clinical trials (RCTs) that compared febuxostat to control groups including placebo and allopurinol. We calculated the pooled relative risk (RR) of MACE and cardiovascular disease (CVD) mortality with the corresponding 95% confidence intervals (CI).

**Results:**

Our search yielded 374 potentially relevant studies. Among the 25 RCTs included in the systematic review, 10 qualified for the meta-analysis. Among the 14,402 subjects included, the median age was 54 years (IQR 52-67) and 90% were male (IQR 82-96); 8602 received febuxostat, 5118 allopurinol, and 643 placebo. The pooled RR of MACE for febuxostat was 0.9; 95% CI 0.6-1.5 (p= 0.96) compared to the control. The RR of CV-related death for febuxostat was 1.29; 95% CI 1.01-1.66 (p=0.03).

**Conclusions:**

Compared with other SU-lowering treatments, febuxostat does not increase or decrease the risk of cardiovascular disease but may increase the risk of CVD death. More RCTs measuring cardiovascular safety as a primary outcome are needed to adequately evaluate the risk of CVD with febuxostat.

## 1. Introduction

Gout is an independent predictor of cardiovascular diseases [1] and serum urate (SU) is associated with the incidence of atrial fibrillation [2, 3], heart failure mortality [4], coronary artery disease calcification [5], and other cardiovascular diseases. However, improving cardiovascular clinical outcomes by reducing SU is controversial [6, 7].

Febuxostat is a nonpurine-like xanthine oxidase inhibitor used in the management of hyperuricemia. Studies have shown no inferiority in reducing SU compared to allopurinol, the most commonly used SU-lowering agent [8]. However, on November 15, 2017, the FDA issued an alert regarding febuxostat [9] based on preliminary results of the CARES trial, conducted in over 6000 patients to assess the cardiovascular safety of febuxostat compared with allopurinol. The full study was subsequently published [10] and showed no difference in overall cardiovascular events, but an increased risk of all-cause and cardiovascular mortality in the febuxostat group.

Twelve systematic reviews or meta-analysis have been published about SU-lowering therapy and gout or hyperuricemia. Six evaluated the efficacy of medications with no mention of cardiovascular events [8, 11–15], three evaluated their renoprotective effects [16–18], one evaluated the effect of SU-lowering therapy on cardiovascular prevention [7], and one evaluated the effect of SU-lowering therapy on the effects on blood pressure [19]. Only one meta-analysis [20] evaluated the cardiovascular events of febuxostat and found no excess events; however, this study did not include CARES, only searched in one database to identify cardiovascular events as adverse events, and only included four studies.

To evaluate the available evidence regarding the cardiovascular safety of febuxostat, we performed a systematic review and meta-analysis of studies comparing hyperuricemic subjects with and/or without gout receiving febuxostat, other SU-lowering therapies, or placebo.

## 2. Materials and Methods

### 2.1. Protocol and Registration

We performed our systematic review according to the Cochrane methodology. The protocol of the present study was not registered. Our report adheres to the Preferred Reporting Items for Systematic Reviews and Meta-Analyses (PRISMA) statement.

### 2.2. Search Strategy

We conducted a literature search using the MEDLINE database through PubMed from inception to 2018. We filtered all articles, except for those containing key terms such as hyperuricemia and febuxostat. We used the following search terms: ＇＇febuxostat＇＇[MeSH Terms] OR ＇＇febuxostat＇＇[All Fields]) AND (＇＇hyperuricaemia＇＇[All Fields] OR ＇＇hyperuricemia＇＇[MeSH Terms] OR ＇＇hyperuricemia＇＇[All Fields])) OR ＇＇Gout＇＇[Mesh] AND ((Clinical Trial[ptyp] OR Clinical Trial, Phase I[ptyp] OR Clinical Trial, Phase II[ptyp] OR Clinical Trial, Phase III[ptyp] OR Clinical Trial, Phase IV[ptyp]) AND ＇＇humans＇＇[MeSH Terms])”. We conducted all searches in March 2018 and supplemented the initial results with manual searches of bibliographies of key relevant articles. In addition, we also searched the EMBASE database using the same timeframe and criteria as well as a search of the clinical trials.gov database for unpublished studies or data on CV events not reported in the original publication. We excluded conference abstracts and articles not published in English because of inability to obtain all the necessary information.

### 2.3. Inclusion and Exclusion Criteria

We included randomized clinical trials that compared febuxostat to one or more control groups, reported adverse events including cardiovascular events, had a follow-up period of any length, and were published in English. We excluded reviews, cohort and cross-sectional studies, and case reports.

### 2.4. Selection Criteria

Two investigators (JC and JB) reviewed the abstract of each citation and identified abstracts for full-text review. When either selected an article for full-text review, it was evaluated by both investigators. Agreement on whether to examine the full text or to incorporate the article in the evidence table was calculated by using interrater agreement.

### 2.5. Data Collection

One investigator (JC) was responsible for completing the evidence table and a second investigator (JB) verified the accuracy of the data collected. Differences between the two final reviewers were resolved by consensus with the senior investigator (LT). From each study we collected the population, indication for the use of SU-lowering medications, demographics, concomitant medications used in the trial, follow-up time, the prevalence of CV risk factors, the number of gout flares, and the incidence of CV events during the follow-up period.

### 2.6. Qualitative Analysis

Because we included only randomized controlled trials we used the 11-item Critical Appraisal Skills program checklist to evaluate the quality of each study [45]. To better describe possible sources of bias, we report on key quality metrics: concealment of allocation, loss to follow-up, cross-over, intention to treat analysis, success of randomization, sample size calculation, missing data, recall bias, and measurement bias. Two investigators were responsible for completing the quality evaluation (JC and JB). Differences between the reviewers were resolved by consensus (led by LT) and we calculated interrater agreement.

### 2.7. Medication Groups

Our main medication of interest was febuxostat. We compared the effect of febuxostat to control groups defined as placebo, allopurinol or other SU-lowering drugs including topiroxostat and benzbromarone. The rationale for grouping placebo and all other medications as one control group was because of the small number of placebo controlled trials (n=4); however we did conduct a sensitivity analysis including only studies that compared febuxostat and placebo. We did not include lesinurad in our analyses, as it is approved only for use with allopurinol or febuxostat and we could not evaluate the individual effect of the medication. To evaluate whether febuxostat dosage was associated with CV events, we classified all studies as either high dose (≥80 mg) or low dose (<80 mg). We selected this cutoff as a previous meta-analysis on the efficacy of the medication used the same cutoffs [8].

### 2.8. Primary and Secondary Outcome

Our primary outcome was major adverse cardiovascular events (MACE). We defined MACE as nonfatal myocardial infarction (MI), angina pectoris, heart failure, ischemic coronary artery disorders, and cardiovascular related deaths [21]. To evaluate the impact of febuxostat on individual outcomes we conducted an analysis on secondary outcomes defined as ischemic heart disease (including unstable angina with coronary revascularization), nonfatal myocardial infarction, and CV-related death.

For our quantitative analysis we only included 10 studies that reported either the primary or secondary outcome we defined previously; the remaining studies were not included in the quantitative analysis.

### 2.9. Subgroup Analysis

To evaluate if the results were impacted by confounders we collected the following information from each study: the study included hyperuricemic patients or only gout patients; concomitant medications that could affect the cardiovascular risk [46] like nonsteroidal anti-inflammatories (NSAIDs) and aspirin; baseline cardiovascular risk including coronary artery disease and cardiovascular risk factors; and the number of gout flares in the febuxostat group as a marker of inflammation. We defined a study as evaluating gout if all patients included in the study had gout and we defined hyperuricemia as those studies that included subjects based on elevations of SU only. We also reported the timing of the cardiovascular (CV) events by study.

### 2.10. Statistical Analysis

We reported relevant baseline characteristics as median values with the interquartile range (IQR). To assess for heterogeneity across studies, we used the Cochran Q chi-square statistic and the I-squared statistic. We defined heterogeneity as an I-squared greater than 50%.

For the quantitative analysis we used Stata 14 (StataCorp LP, College Station, TX). We evaluated publication bias using the Egger's method. We calculated the pooled relative risk (RR) of the primary and secondary outcome with the respective 95% confidence intervals (CIs) for febuxostat compared to the control arm using the fixed effects method.

We also conducted metaregression for the primary and secondary outcome as the dependent variable [47] and then calculated the exponentiated beta-coefficient using the random effects model and included baseline cardiovascular risk, follow-up time, gout versus hyperuricemia, NSAID or aspirin use, and the number of gout flares in the model.

## 3. Results

### 3.1. Literature Search

**Figure 1:**
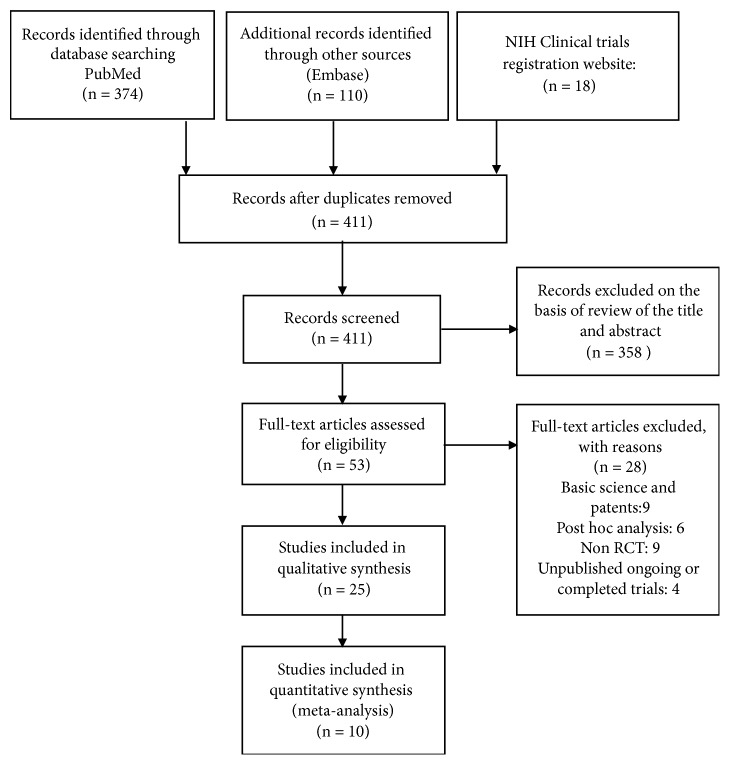
*Flowchart of included studies*. NIH: National Institutes of Health. RCT: randomized clinical trial.

Figure 1 shows the flowchart of included and excluded studies. Our search yielded 374 abstracts from PubMed, 110 from EMBASE, and 18 protocols from the clinicaltrials.gov database. From a total of 502 citations we removed 91 duplicate abstracts. We excluded 358 studies at the abstract level and selected 53 for full-text review; of these we excluded 28. Therefore, 25 studies were finally included. Interrater agreement between reviewers on inclusion versus exclusion of studies was 100%. From the clinicaltrials.gov register, we retrieved additional cardiovascular events data for 5 of the 25 published studies [25, 38–41]. Among the 25 included studies, 10 reported MACE and 15 reported no CV events during the study follow-up.

### 3.2. Baseline Characteristics

Table 1 shows the baseline characteristics of the 25 studies included in the qualitative review. The studies included 14,402 participants with hyperuricemia: 8,602 (59%) received febuxostat, 5,118 (36%) allopurinol, and 643 (4%) placebo. The median age of the trial participants was 54 years (IQR 52-67) and 90% (IQR 82-96) were male. The median follow-up of the studies was 6 months (IQR 2.7-12), 53% (IQR 43-92) had hypertension, and 32% (IQR 11-38) had diabetes.

Studies that reported CV events had a median follow-up of 12 months (IQR 6.4-24) compared to studies that reported no CV events in which the median follow-up was 3.6 months (IQR 1.8-6). Nine of the 15 studies that reported no CV events evaluated febuxostat on several cardiovascular conditions.

Only ten of the studies were exclusively conducted in North America. Twelve of the studies compared febuxostat to allopurinol. The remaining studies compared febuxostat to placebo (10), to both placebo and allopurinol (1), to topiroxostat (n=1), and to benzbromarone (n=1).

Eleven studies included patients with gout exclusively, 5 recruited patients with hyperuricemia and no evidence of gout, and the remaining 9 studies included patients with cardiovascular diseases.

### 3.3. Qualitative Evaluation

The beta-coefficient for publication bias was 0.30; 95% CI -0.81-1.42, p=0.53. Our qualitative evaluation found that all but three studies were blinded. Outcomes other than SU were infrequently reported, particularly in short follow-up studies designed to evaluate safety of febuxostat on patients with CV risk factors. The CARES trial was the only study designed to evaluate the long-term cardiovascular safety of febuxostat as the main endpoint. Despite its relevance, the CARES trial had a significant limitation, almost 57% of patients discontinued the trial treatment prematurely, and 45% of the participants did not complete follow-up which could have caused a considerable impact on the results. However, the number of participants who discontinued the trial and did not complete the follow-up was similar in both arms of the study.

### 3.4. Association between Febuxostat and MACE

**Figure 2:**
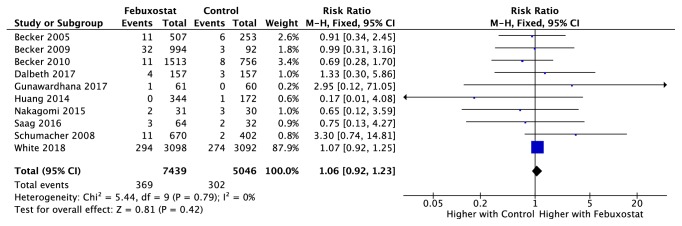
Forest plot of the association between MACE and febuxostat.

Ten studies (Table 2) reported the association between febuxostat and MACE [10, 21, 22, 32, 38–41, 43, 44]. Nine of these studies included patients with a recorded history of either hypertension, heart failure, or coronary artery disorder, whereas the CARES trial had the higher proportion.  Figure 2 shows the association between febuxostat and MACE compared to control groups. The RR of MACE for any febuxostat dosage was 1.06; 95% CI 0.92-1.23 (p=0.42) with an I-squared of 0%. The RR of MACE for any febuxostat dosage when the control group was placebo was 1.81; 95% CI 0.72-4.53 (p=0.20). The RR of MACE for low dose febuxostat dosage was 1.03; 95% CI 0.65-1.66 (p=0.43). The RR of MACE for high dose febuxostat dosage was 0.98; 95% CI 0.47-2.04 (p=0.97). Only two studies reported the timing of the CV events and most of them occurred within 24 months of exposure to the medication.

### 3.5. Association between Febuxostat and the Secondary Outcome

Six studies reported the secondary outcome [10, 21, 38, 39, 41, 44]. The RR of CV-related death for febuxostat was 1.29; 95% CI 1.01-1.66, p=0.03. When the largest study (CARES) [10] was excluded, the RR of CV death for febuxostat was 0.73; 95% CI 0.24-2.25, p=0.64.

### 3.6. Subgroup Analysis

For the primary outcome, one has the following: the metaregression beta-coefficients for gout (1.63), number of gout flares (1.00), coronary artery disease (1.00), follow-up time (1.00), NSAID (0.99), and aspirin (1.00), all with a p-value>0.05. For the secondary outcome, one has the following: the metaregression beta-coefficients for coronary artery disease (1.70), number of gout flares (1.00), follow-up time (1.00), NSAID (1.02), and aspirin (0.98), all with a p-value>0.05.

## 4. Discussion

Our study found that febuxostat does not increase the overall risk of major adverse cardiovascular events among patients with gout and hyperuricemia. However, our analysis also showed an increased the risk of cardiovascular death with febuxostat. We did not find that CAD, NSAID or aspirin use, number of gout flares, or including only gout patients were factors related to the excess cardiovascular death.

Elevated SU is associated with increasing heart failure incidence [48, 49], heart failure mortality [4], incident hypertension [50], incident atrial fibrillation [3], and ischemic heart disease [51]. This finding has made SU a therapeutic target to improve cardiovascular outcomes by modifying the nitroso/redox balance; however, allopurinol has not improved outcomes in heart failure [6, 52]. Therefore, there is a need for newer medications that can affect the nitroso/redox balance.

The FDA required Takeda Pharmaceuticals [9], to conduct a cardiovascular safety clinical trial based on clinical trial results [39, 40] and observational data [53] showing a possible excess in cardiovascular events before its approval in 2009. On November 2017, the FDA released a safety alert based on the results of the Cardiovascular Safety of Febuxostat or Allopurinol in Patients with Gout trial (CARES trial). The study had a randomized double-blind design and included patients with gout and a history of major cardiovascular disease and found no increase in the primary outcome of MACE, defined in this RCT as CV-related death, nonfatal MI, nonfatal stroke, and unstable angina requiring urgent revascularization, but found an increase in the risk of the secondary endpoints of cardiovascular death and death from all causes in intention to treat and per protocol analyses. Due to the CARES specific design and being the only RCT published focused on measuring the febuxostat cardiovascular safety, it constitutes over 80% of our pooled analysis.

There are several potential explanations for our findings and those of the CARES trial. First, the use of NSAIDs has been associated with increasing rates of cardiovascular outcomes [54], and it is possible that NSAID use for gout prophylaxis and/or treatment may have skewed the data. While we did not observe an NSAID effect, NSAIDs were not well documented in the included studies and may have had unmeasured effects. Second, gout flares and the inflammation they entail have been associated with myocardial infarction [55]. However, in CARES, NSAID use and gout flares were similar between those using febuxostat and allopurinol. Colchicine, which has been suggested to lower rather than raise cardiovascular risk, is also used for both gout prophylaxis and acute treatment, but the data available in the studies did not permit rigorous analysis of whether colchicine use could have biased our outcomes. Third, xanthine oxidase inhibition could disrupt the activity of nitric oxide synthase [56] which can in turn reduce the coronary blood flow and cause ineffective excitation contraction myocardial coupling [6, 56]. Paradoxically, however, urate itself has also been shown to inhibit the ability of endothelial cells to secrete nitric oxide [57, 58]. The nature of our meta-analysis did not permit us to assess the impact and balance of the relative effects of nitric oxide inhibition and SU-lowering on coronary blood flow.

Our study also found that several studies were conducted in different CV at risk populations but had short follow-up and they were unable to identify CV events. All of these studies were funded by the makers of febuxostat. Typically, phase 2 and 3 studies can identify common safety issues but may not be of sufficient scope or duration to identify important but less common events.

The present study was unable to identify a profile of risk since factors included in our metaregression analysis were nonsignificant. However, the highest coefficients of risk were seen with the presence of coronary artery disease at baseline and with those studies that exclusively tested febuxostat on gout patients.

The strengths of our study include the extensive search for cardiovascular events, the inclusion only of randomized clinical trials, the number of studies, and the evaluation of potential explanations for the excess risk.

Despite the strengths, our study has important limitations that deserve to be mentioned. First, despite having a considerably high number of studies and patients within those studies, the number of MACE or any cardiovascular event was low in both groups. This could be explained by a low incidence of risk or by the fact that cardiovascular safety was not the primary endpoint in all but one of the included trials [10], along with the short follow-up of most studies. Second, the exclusion criteria in some studies prevented patients with a history of cardiovascular disease to enter in their studies, thus reducing the patients at risk of developing MACE. Third, not all studies consistently reported the data about NSAID and aspirin use and the cardiovascular level of risk of the included patients, therefore, limiting or metaregression. Fourth, our findings of increasing CV-related death were driven by the large sample size and event rate of the CARES trial [10]. Finally, the potentially important question raised by the CARES trial, of whether febuxostat may independently increase risk of CV-related death or decrease the risk of CV-related death but to a lesser extent than allopurinol, could not be determined, given our methodology and the available data.

In conclusion, our meta-analysis of available studies indicates that while febuxostat neither increases nor decreases the risk of major adverse cardiovascular outcomes in hyperuricemic patients, it may increase the risk of CV-related death in patients with gout and a history of prior CV events. Given the high prevalence and the chronic nature of gout, caution is appropriate when deciding whether to use febuxostat in high-risk CV patients. More RCTs measuring cardiovascular safety as a primary outcome are needed to adequately evaluate the risk of CVD with febuxostat.

## Figures and Tables

**Table 1:** Baseline characteristics.

Author	Country	Population	n	Age (SD)	Males %	Intervention	Control group	Follow up (m)	Industry funding	Gout	HTN	CAD	HF	T2DM
White [10]	US	Gout + previous CV events	6190	65 IQR (58-71)	84	FBX 40 mg up to 80 mg	Allopurinol	32	Yes	100	92	76	-	20

Dalbeth [21]	US	Gout	314	51 (+/-12)	92	FBX 40 mg/ 60 mg/ 80 mg	Placebo	24	Yes	100	-	-	-	-

Gunawardha [22]	US	HTN	121	54 (+/-10.6)	81	FBX 80 mg	Placebo	1.5	Yes	-	100	-	-	-

Sezai [23]	Japan	CVD	55	68 (+/-9)	83	FBX 60 mg	Topiroxostat	12	Yes	-	82	36	-	35

Ohta [24]	Japan	HTN	20	64 (+/-12)	90	FBX 40 mg	Benzbromarone	9	No	-	100	-	-	-

Beddhu [25]	US	CKD T2DM	80	68 (+/-10)	65	FBX 80 mg	Placebo	5.5	Yes	-	77	18	10	100

K-H [26]	China	Gout	109	45 (+/-11.5)	97	FBX 80 mg	Allopurinol	2.7	Yes	100	-	-	-	-

Sircar [27]	India	CKD	108	57 (+/12.6)	70	FBX 40 mg	Placebo	6	Yes	-	98	38	-	38

Tani [28]	Japan	HTN	60	67 (+/-12)	88	FBX 20 mg	Placebo	6	No	-	100	32	-	32

Tanaka [29]	Japan	CKD	45	68 (+/-8.2)	88	FBX 40 mg	Allopurinol	2.7	No	-	43	-	-	-

Tsuruta [30]	Japan	ESRD	53	68 (+/-12.55)	64	FBX 10 mg	Placebo	1	No	-	79	27	-	42

Xu [31]	China	Gout	504	47 (+/-11.5)	95	FBX 40 mg/ 80 mg	Allopurinol	5.5	Yes	100	16	-	-	5

Huang [32]	China	Gout	512	47 (+/-11.2)	98	FBX 40 mg/ 80 mg	Allopurinol	6.4	Yes	100	28	-	-	-

Sezai [33]	Japan	Cardiac surgery	141	67 (+/-10.3)	82	FBX 60 mg	Allopurinol	6	No	-	81	40	-	36

Katamani [34]	Japan	Hyperuricemia	40	54 (+/-10.4)	100	FBX 40 mg/ 60 mg	Allopurinol	3.6	Yes	76	42	-	-	5

Katamani [35]	Japan	Hyperuricemia	202	53 (+/-12.7)	97	FBX 20 mg/ 40 mg/ 60 mg/ 80 mg	Placebo	3.6	Yes	59	49	-	-	11

Katamani [36]	Japan	Hyperuricemia	102	48 (+/-13.6)	100	FBX 20 mg/ 40 mg	Placebo	1.8	Yes	49	35	-	-	11

Katamani [37]	Japan	Hyperuricemia	244	52 (+/-13.5)	98	FBX 40 mg	Allopurinol	1.8	Yes	47	33	-	-	10

Becker [38]	US	Gout	2269	52 (+/-11.7)	94	FBX 40 mg/ 80 mg	Allopurinol	6	Yes	100	53	-	-	14

Becker [39]	US	Gout	1086	51 (+/-11.6)	96	FBX 80 mg/ 120 mg	Allopurinol	40	Yes	100	45	-	1	7

Schumacher [40]	US	Gout	1072	52 (+/-12.2)	94	FBX 80 mg/ 120 mg/ 240 mg	Allopurinol and placebo	6.4	Yes	100	47	-	-	-

Becker [41]	US	Gout	762	52 (+/-12.1)	96	FBX 80 mg/ 120 mg	Allopurinol	12	Yes	100	44	10	-	7

Becker [42]	US	Gout	153	54 (+/-12.6)	89	FBX 40 mg/ 80 mg/ 120 mg	Placebo	1	Yes	100	49	60	-	13

Nakagomi [43]	Japan	HF+Hyperuricemia	61	70.5 (+/-9)	70	FBX 40 mg	Allopurinol	12	No	-	93	72	100	34

Saag [44]	US	Gout+CKD	96	65.7 (+/-10)	80	FBX up to 80 mg	Placebo	12	Yes	100	95	-	-	44

n: number of participants. HTN: hypertension. CAD: coronary artery disease. HF: heart failure. T2DM: type 2 diabetes mellitus. CV events: cardiovascular events. CKD: chronic kidney disease. ESRD: end-stage renal disease. SD: standard deviation. IQR: interquartile range. FBX: febuxostat.

**Table 2:** Major adverse cardiac events.

Author	Drug	n	Angina pectoris	HF	Ischemic Coronary artery disorder	Non-fatal MI	CV death	MACE
Dalbeth [21]	FBX 40 mg	157	0	1	1	1	1	4
	Placebo	157	0	1	1	0	1	3

Gunawardhana [22]	FBX 80 mg	61	0	0	1	0	0	1
	Placebo	60	0	0	0	0	0	0

Huang [32]	FBX 40 mg	172	0	0	0	0	0	0
	FBX 80 mg	172	0	0	0	0	0	0
	Allopurinol	172	1	0	0	0	0	1

Becker [38]	FBX 40 mg	757	2	2	4	0	0	8
	FBX 80 mg	756	0	0	2	1	0	3
	Allopurinol	756	0	1	4	1	2	8

Becker [39]	FBX 80 mg	606	0	3	17	0	4	24
	FBX 120 mg	388	0	3	3	0	2	8
	Allopurinol	92	0	1	2	0	0	3

Schumacher [40]	FBX 80 mg	267	0	0	3	0	0	**5** **∗**
	FBX 120 mg	269	0	0	2	0	0	**5** **∗**
	FBX 240 mg	134	0	0	0	0	0	**1** **∗**
	Allopurinol	268	0	0	0	0	0	**1** **∗**
	Placebo	134	0	0	1	0	0	**1** **∗**

Becker [41]	FBX 80 mg	256	0	3	3	0	1	7
	FBX 120 mg	251	0	1	2	0	1	4
	Allopurinol	253	0	0	6	0	0	6

Nakagomi [43]	FBX 40	31	0	0	2	0	0	2
	Allopurinol	30	0	0	3	0	0	3

Saag [44]	FBX 30 mg BID	32	0	1	0	0	0	1
	FBX 48/80 mg QD	32	0	1	0	0	1	2
	Placebo	32	0	0	0	1	1	2

White [10]	FBX up to 80	3098	0	0	49	111	134	294
	Allopurinol	3092	0	0	56	118	100	274

FBX: febuxostat; HF: heart failure; MACE: major adverse cardiovascular events. **∗**Reported as cardiovascular events (chest pain, coronary artery disease, myocardial infarction, and atrial fibrillation), not specified how many of each.

## Data Availability

The data points used to support the findings of this study are included within the article.
